# The RNAi Mechanism Regulates a New Exonuclease Gene Involved in the Virulence of Mucorales

**DOI:** 10.3390/ijms22052282

**Published:** 2021-02-25

**Authors:** Carlos Pérez-Arques, María Isabel Navarro-Mendoza, Laura Murcia, Eusebio Navarro, Victoriano Garre, Francisco Esteban Nicolás

**Affiliations:** Departmento de Genética y Microbiología, Facultad de Biología, Universidad de Murcia, 30100 Murcia, Spain; carlos.parq@duke.edu (C.P.-A.); maribel.navarro@duke.edu (M.I.N.-M.); lauramur@um.es (L.M.); sebi@um.es (E.N.)

**Keywords:** mucormycosis, *Mucor lusitanicus*, Mucorales, RNAi, exonuclease, *wex1* gene, virulence, virulence factor

## Abstract

Mucormycosis is a lethal disease caused by Mucorales, which are emerging as human causes that explain the high mortality for this disease. Consequently, the research community is searching for virulence determinants that could be repurposed as targets to develop new treatments against mucormycosis. Our work explores an RNA interference (RNAi)-based approach to find targets involved in the virulence of Mucorales. A transcriptomewide analysis compared sRNAs and their target mRNAs in two *Mucor lusitanicus* different pathotypes, virulent and avirulent, generating a list of 75 loci selected by their differential sRNA accumulation in these strains. As a proof of concept and validity, an experimental approach characterized two loci showing opposite behavior, confirming that RNAi activity causes their differential expression in the two pathotypes. We generated deletion mutants for two loci and a knockin-strain overexpressing for one of these loci. Their functional analysis in murine virulence assays identified the gene *wex1,* a putative DEDDy exonuclease with RNase domains, as an essential factor for virulence. The identification of *wex1* showed the potential of our approach to discover virulence factors not only in Mucorales but also in any other fungal model with an active RNAi machinery. More importantly, it adds a new layer to the biological processes controlled by RNAi in *M. lusitanicus*, confirming that the Dicer-dependent RNAi pathway can silence gene expression to promote virulence.

## 1. Introduction

Pathogens and hosts are continuously adapting their defense mechanisms to succeed and survive during their interactions. Among human fungal pathogens, Mucorales are emerging as a source of new adaptations, such as their capability of infecting immunocompetent hosts, general resistance to most of the current antifungal compounds, and aggressive and virulent behavior during the infection known as mucormycosis [1,2,3,4,5,6] These potentiated virulence features can explain our current lack of effective treatments, which is the direct cause behind the alarming mortality rates that exceed 90% in disseminated infections [7,8,9]. In this context, we are a step behind in the fight against these emerging pathogens, evidencing the imperious demand for studies designed to discover new molecular targets that can serve to develop effective antifungal compounds.

The main reason explaining the lack of targets for effective antifungal compounds is the disparity between Mucorales and other distant fungi like Ascomycetes and Basidiomycetes [10,11]. Thus, our arsenal of antifungal compounds was initially selected for their high efficiency against other fungal pathogens such as *Aspergillus* and *Candida* spp., neglecting the study of mucormycosis due to it being considered a rare infection only affecting immunocompromised patients until very recently [3,12]. Mucoralean pathogens possess specific attributes like nonseptated hyphae and a cell wall containing the polysaccharide chitosan, an N-deacetylated version of chitin [10]. Recently, new studies have found other molecular divergences as their atypical centromeric chromatin, which have lost two essential components of the kinetochore—CENP-A and CENP-C—resulting in a mosaic of point and regional centromeres [13]. Regarding their intrinsic antifungal drug resistance, the dissection of the enzymes involved in the synthesis of ergosterol has unveiled another deviation only conserved in these fungi. Thus, the lanosterol 14α-demethylase CYP51 F5 of Mucorales has two amino acid substitutions compared to other fungi, which has been related to their resistance to short-tailed azole compounds [4]. However, there is a molecular process that has paramount importance in regulating the biology of these fungi: their RNAi mechanism. In Mucorales, RNAi has been related to their antifungal resistance and virulence, but also to their general physiology [6,14,15]. Up to three different RNAi pathways have been described and involved in the gene regulation of the fungus *Mucor lusitanicus* [16,17]. The first one produces small RNAs from exonic regions (ex-siRNAs, exonic small interfering RNAs) that regulate hundreds of mRNAs [18]. Mutations in essential genes of this pathway show defects in general processes such as growth, morphology, asexual sporulation, and sexual reproduction [14]. The second pathway, known as the epimutation pathway, is directly involved in an exclusive antifungal resistance mechanism of Mucorales [6,19,20]. This pathway generates strains which are temporarily resistant to antifungal compounds like FK506 and 5-fluoroorotic acid (5-FOA). This transient resistance is lost as soon as the antifungal compound is removed from the media. The mechanism behind this resistance is RNAi, which explicitly degrades the mRNA of the genes that are targets of the antifungal compounds. Thus, the RNAi mechanism specifically degrades the mRNAs of pyrimidine synthesis genes −*pyrG* or *pyrF*− in the presence of 5-FOA, whereas the *fkbA* mRNA is degraded in the presence of FK506. The third RNAi pathway is the most recently described in Mucorales and also one of their unique features as it is only conserved in this group of fungi [21]. It is a noncanonical RNAi pathway (NCRIP) because its machinery does not depend on Dicer and Argonaute proteins to degrade transcripts. Instead, NCRIP relies on RNA-dependent RNA polymerases (RdRPs) and a novel ribonuclease III-like named R3B2. NCRIP is involved in the regulation of virulence in Mucorales. Thus, mutants in this pathway show reduced virulence in a murine model, likely because NCRIP regulates the expression of hundreds of genes during the interaction of macrophages and fungal spores [22,23,24,25].

The studies dissecting the RNAi pathways of Mucorales have unveiled the regulation of thousands of genes by this mechanism. Although many of these genes must be involved in virulence, the broad regulatory control of RNAi over other physiological processes hinders the identification of unknown virulence factors. Previous works demonstrated the validity of a strategy based on the comparative genomic analysis between virulent and avirulent strains of *Mucor lusitanicus* [26]. Focusing on these different pathotypes, a comparative transcriptional analysis during macrophage phagocytosis revealed an intricate gene network activated in the virulent strain to survive the immune response [22]. Here, we propose a similar approach but comparing the loci regulated by RNAi in the virulent and avirulent pathotypes. The result of this strategy revealed a population of differentially regulated loci accumulating sRNAs that could be involved in the virulence differences observed in these strains. Selected candidate genes from this population have been experimentally evaluated, demonstrating that our strategy can identify new virulence factors regulated by RNAi. 

## 2. Results

### 2.1. Comparative Analysis of sRNAs Transcriptomic Profiles between Virulent and Avirulent Pathotypes

**Figure 1:**
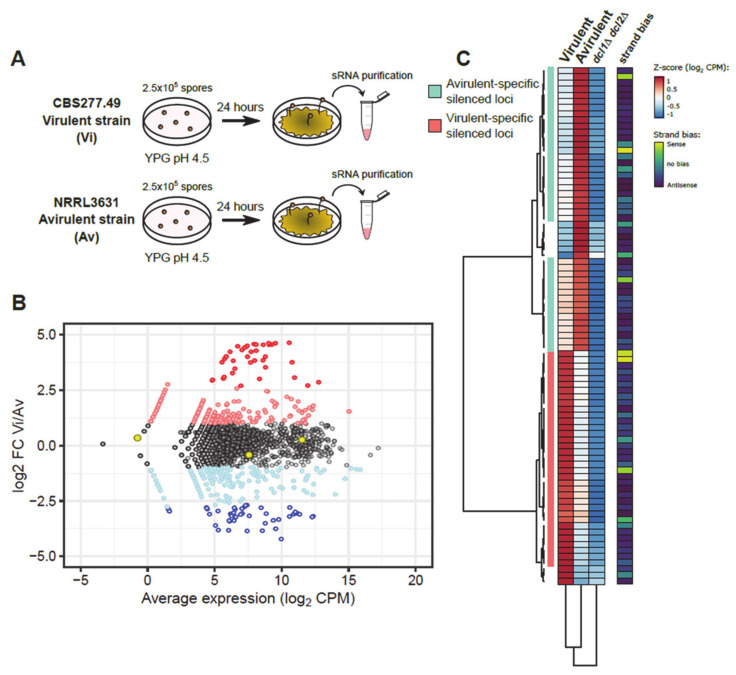
RNAi machinery targets different subsets of genes in each pathotype. (**A**) Experimental design for sRNA purification from two different pathotypes of *M. lusitanicus*: strains CBS277.49 and NRRL3631, respectively. (**B**) Scatterplot showing differences in sRNA accumulation in both pathotypes, virulent (Vi) and avirulent (Av). Each dot represents a protein-coding locus of the annotated *M. lusitanicus* genome (Muccir1_3), showing the log_2_ fold-change (FC) between Vi and Av (y-axis) and the average expression in both pathotypes (x-axis, in log_2_ CPM). Each gene is color-coded to depict nonsignificant FC (black), upregulation (pink, log_2_ FC ≥ 1) or downregulation (light blue, log_2_ FC ≤ −1), and significant upregulation (red, log_2_ FC ≥ 2.5 and *p*-value ≤ 0.05) or downregulation (blue, log_2_ FC ≥ -2.5 and *p*-value ≤ 0.05). Three housekeeping genes are marked as yellow to assure a proper normalization among samples. (**C**) Heatmap showing the accumulation of sRNAs (calculated as the Z-score of log_2_ CPM) in 84 protein-coding loci that showed significant log_2_ FC differences between Vi and Av. Equivalent values in an RNAi-deficient *dcl1* and *dcl2* double deletion mutant (*dcl1*Δ *dcl2*Δ) are shown. Values are clustered by similarity in protein-coding loci (rows) and strains (columns), identifying two major subsets of protein-coding loci. Strand bias, i.e., the proportion of antisense: sense sRNAs are shown in the rightmost column.

Previous studies showed differential phenotypes regarding virulence between two opposite mating type strains of *Mucor lusitanicus*. Thus, the mating-type (−) CBS277.49 and its derivative strains (hereinafter Vi) have an increased virulence potential in immunosuppressed mice, manifested by high mortality rates. In opposition, the mating-type (+) NRRL3631 (hereinafter Av) is harmless at the same doses [26]. Furthermore, several differences at a genomic and transcriptomic level were related to the opposite virulent behavior of these two strains [26]. However, the complexity of the pathogenic potential and the profuse regulatory functions of the RNAi mechanism in *M. lusitanicus* suggested an extra regulatory layer at the transcriptomic level [22]. To test this hypothesis and explore the role of the RNAi mechanism in the virulence of Mucorales, sRNA samples from both strains were deeply sequenced and compared (Figure 1A). sRNA samples were obtained from mycelia grown for 24 h at 26 °C in rich medium YPG. After sRNA sequencing, sRNA reads were mapped to the genome of *M. lusitanicus* (Muccir1_3), to compare the endogenous short RNA (esRNA) accumulation patterns between the two pathotypes. The results of this analysis revealed a total of 843 loci showing differential esRNA accumulation (log_2_ FC ≥ 1 and ≤ −1) (Figure 1B), (Table S1). The amount of sRNA overaccumulating loci is evenly distributed among Vi and Av. There are 454 sRNA overproducing loci in the Vi pathotype as opposed to 350 loci in the Av pathotype. Among these loci, we selected a total of 84 showing high significance (log_2_ FC ≥ 2.5 and ≤ −2.5, and *p*-value ≤ 0.05) as the most likely regions regulated by the RNAi pathway (Figure 1B,C), (Table S2). *M. lusitanicus* harbors several RNAi pathways, both Dicer-dependent and independent. To discriminate between the two, the transcriptomic sRNA data of a double Dicer mutant (*dcl1*Δ *dcl2*Δ) were also analyzed. Loci producing Dicer-dependent sRNAs in the Vi or Av pathotype would show a substantial decrease in sRNA production in the *dcl1*Δ *dcl2*Δ mutant, confirming that Dicer activity is needed to generate those sRNAs. A total of 75 loci from the previous 84 show a decrease of sRNAs in this double mutant (Figure 1C, third column) [27,28], suggesting that Dicer-dependent pathways are critical for the differences observed among the two pathotypes. In *M. lusitanicus*, Dicer generates both sense and preferentially antisense sRNAs to promote active silencing. Hence, the average ratio of sense:antisense sRNAs in the two pathotypes was analyzed, confirming that these loci produced mainly antisense sRNAs. To further delimitate the role of the RNAi pathway in the two strains, the 75 loci were grouped in two different clusters: 35 of them showing low accumulation in the Vi strain, and 40 showing low accumulation in the Av strain (Figure 1C, first and second columns, respectively). Considering the different virulence potentials observed between both pathotypes, the sRNA profiles analyzed here led us to identify a specifically delimitated list of candidates to find new genes involved in the pathogenesis of Mucorales. 

### 2.2. Validation of Target Genes That Are Differentially Regulated by RNAi

**Figure 2:**
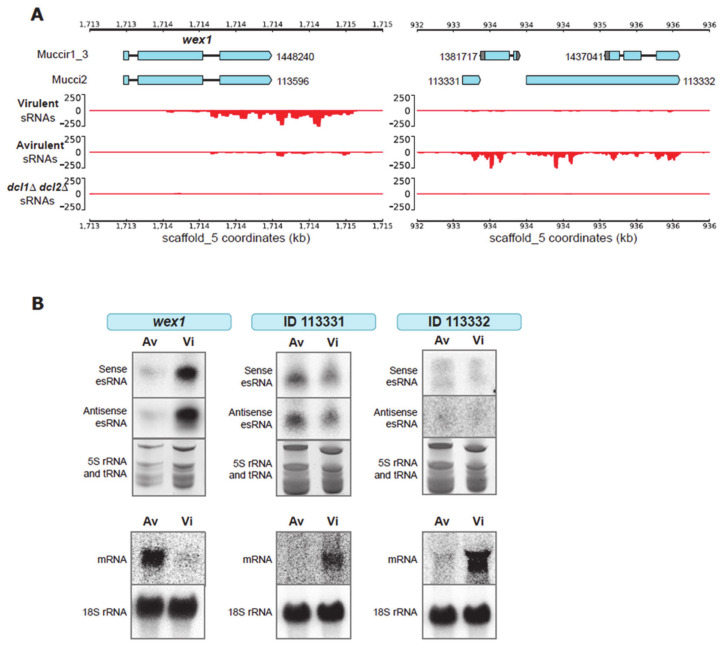
A putative DEDDy exonuclease and a 3-kb genomic region are differentially targeted by RNAi in each pathotype. (**A**) Genomic plots of two selected candidate regions: one containing the *wex1* gene that is actively silenced in the Vi pathotype, and the other spanning a 3-kb sequence that is targeted in the Av pathotype. Genome annotation and gene IDs from two different models (Muccir1_3 filtered models and Mucci2) are shown. Below, each track shows the accumulation of sense (y-axis, positive values) and antisense sRNAs (y-axis, negative values) as CPM values across the genomic coordinates (x-axis). (**B**) The sRNA Northern blots (above) show sense and antisense sRNA accumulation in each strain depicted for three analyzed loci: *wex1*, 113331 and 113332 (from Mucci2). Loading controls consisting of tRNAs and 5S rRNA stained with ethidium bromide are shown. The mRNA Northern blots (below) show the transcription of each analyzed locus in the depicted strains, confirming the inverse correlation between sRNA accumulation and mRNA levels. The expression level of 18S rRNA is shown as a loading control.

The next step in our strategy was to validate the results of the transcriptomic analysis (previous section) by an alternative approach. We randomly selected two loci, one showing low levels specifically in the Vi strain and another one in the Av strain (Figure 2A). The locus selected from the Vi strain contained one gene (ID 148240) encoding a protein with nuclease features. In particular, this gene showed similarity with the Werner Syndromelike exonuclease (WEX) (a DEDDy exonuclease, part of the DnaQ-like exonuclease superfamily), denominating the gene as *wex1* in *M. lusitanicus* [29]. The alignment of the sRNAs showed that an important amount of them were generated from the 3′-UTR region, suggesting a regulatory role of this region. Surprisingly, the locus selected from the Av strain contained several putative genes, interspersed in the same 3-kb esRNA-producing region, indicating that they share the same regulation via the RNAi pathway. These genes are unique in Mucorales, and their function is unknown. This region was analyzed as a whole, denominating the locus as Avirulent-RNAi-Dependent-Locus (ARDL). To confirm RNAi activity, we measured the esRNAs and the mRNA levels in the two pathotypes by Northern blots using specific probes from the gene *wex1* and the locus ARDL. The blots showed elevated production of *wex1* esRNAs and reduced levels of the corresponding mRNA in the Vi strain (Figure 2B) and, conversely, elevated accumulation of ARDL esRNAs and reduced levels of the corresponding mRNA in the Av strain (Figure 2B). These results, along with the lack of sRNAs in the double Dicer mutant profiles, confirmed the differential behavior of RNAi in the Vi and Av pathotypes for the two studied loci.

### 2.3. Generation of Mutant Strains with Altered Expression of the Candidate Genes

**Figure 3:**
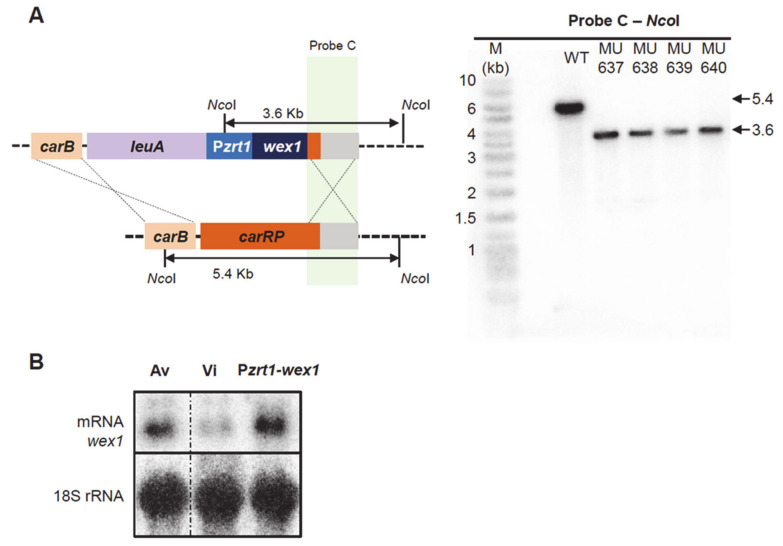
Overexpression of *wex1* overcomes RNAi silencing. (**A**,**B**) Southern blot depicting the generation of the *wex1* overexpressing mutant P*zrt1-wex1*. A schematic representation of the wild-type and mutant loci after homologous recombination with the designed overexpression fragment targeting the *carRP* locus (depicted as crossing lines) is shown to the left. Fragments digested with NcoI and their expected sizes are indicated. The position of the radioactive probe and its complementary sequence is shown. To the right, the blots showing the digested DNA fragments hybridizing to the probe C of each mutant and wild-type strains allow for size discrimination using a DNA ladder (M). (**B**) The transcription of *wex1* is shown in the P*zrt1-wex1* mutant and compared to both Av and Vi pathotypes, measured by Northern blot, and 18S rRNA is shown as a loading control. Samples are rearranged for consistency (shown as a discontinuous line).

**Figure 4:**
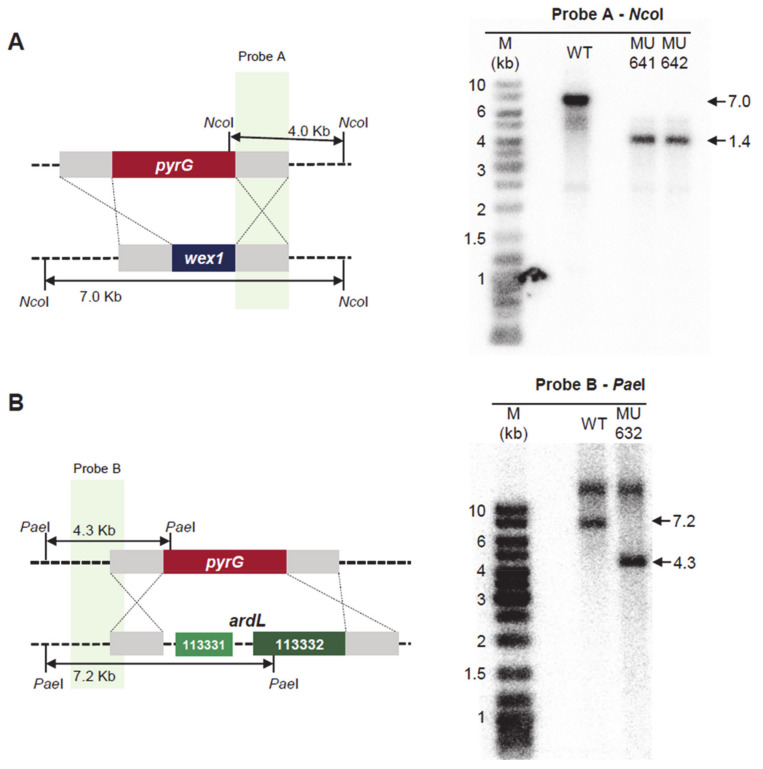
Deletion mutants in *wex1* and ARDL. (**A**,**B**) Southern blots illustrate the generation of (**A**) the *wex1* and (**B**) the ARDL deletion mutants. A schematic representation of the wild-type and mutant loci after homologous recombination with the designed disruption fragment (depicted as crossing lines) is shown to the left. Fragments digested with (**A**) NcoI or (**B**) PaeI and their expected sizes are indicated. Radioactive probes and their complementary sequences are shown in each locus. To the right, the blots showing the digested DNA fragments of each mutant and wild-type strain hybridizing to the corresponding probes allow for size discrimination using a DNA ladder (M).

The RNAi-based functional transcriptomic approach applied to the identification of new virulence factors resulted in a narrowed list of 75 candidates. The validation of this approach to identify virulence factors required the generation of different mutants that mimicked the behavior of the Av pathotype. The *wex1* gene was being targeted by the RNAi degrading mechanism in the Vi pathotype, as opposed to its upregulation in the Av pathotype. Accordingly, we generated a strain that overexpressed *wex1* in the Vi pathotype genetic background to study its role in *M. lusitanicus* virulence. To overexpress this gene, we constructed a knockin vector containing an engineered cassette with adjacent regions of the *carRP* gene, the selectable marker *pyrG* gene, and the *wex1* gene fused to the previously described strong promoter P*zrt1* (Figure 3A, left) [30,31] The integration into the locus *carRP* facilitates the screening for homokaryotic target mutants, as they show an albino phenotype [31]. The proper insertion of this cassette was confirmed in four independent mutants by Southern blot (all of them generated from the virulent strain and named as MU637, MU638, MU639 and MU640) (Figure 3A, right). Analysis of *wex1* expression by Northern blot (Figure 3B) showed an increase of expression of this gene in the knockin strain, showing similar levels to the Av strain. We also generated a deletion mutant for the *wex1* gene, as a control strain, by using a knockout vector designed to replace *wex1* with the *pyrG* gene after homologous recombination. This knockout vector contained an engineered cassette with adjacent regions of the *wex1* gene flanking the *pyrG* gene (Figure 4A, left). The correct insertion of this cassette was also validated in two independent mutants by Southern blot (Figure 4A, right). On the contrary, the locus ARDL was upregulated in Vi compared to the specific RNAi-based degradation and downregulation in the Av strain. To mimic this regulation found in the Av pathotype, we generated a knockout mutant of ARDL in the Vi strain. Following a similar approach to the *wex1* knockout strain, we generated a cassette with the adjacent regions of ARDL flanking the *pyrG* gene (Figure 3B, left), and the insertion of this cassette was confirmed in two independent mutants by Southern blot (Figure 3B, right).

### 2.4. Functional Analysis of the Genes Regulated by RNAi and Their Role in Virulence

**Figure 5:**
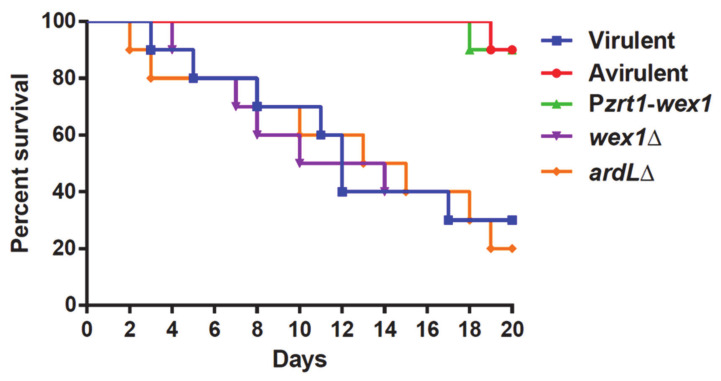
Silencing *wex1* is critical for virulence. Virulence assays in immunosuppressed mice injected with 1 × 10^6^ spores of the *wex1*Δ (purple) and P*zrt1*-*wex1* (green, (MU637)) mutants. As infection controls, the mice were also injected with spores from the recipient transformation strain R7B that derives from the Vi pathotype (blue) and the Av pathotype (red). The survival rate of the mutants was compared to the Vi control strain and statistically analyzed by a Mantel—Cox test.

The virulence of fungal pathogens is an intricate feature depending on multiple cellular processes, mechanisms, and gene pathways [32]. The final aim of this work was to find new target genes both regulated by RNAi and related to pathogenesis in Mucorales. Thus, the *wex1* knockin and ARDL deletion mutants were studied in a virulence assay employing a mouse model previously validated in *M. lusitanicus* [7,33]. As control strains, the *wex1* deletion mutant and the Vi and Av pathotypes were also injected in mice. The overexpression of *wex1* in the knockin mutant resulted in an avirulent phenotype like the Av pathotype, whereas the virulence potential of the mutant *wex1*Δ was not affected (Figure 5). These results indicate that RNAi silencing of *wex1* in the Vi pathotype is essential to uphold its virulence potential. The overexpression of *wex1* probably compensates for the RNAi degradation, mimicking the transcription levels of the Av strain. The deletion of ARDL in Vi simulated the silencing of this locus observed in the Av strain; however, this mutation did not alter the virulence. These results suggest that the ARDL region has a function nonrelated to virulence, although it might be involved in other cellular processes that differ between the Vi and Av pathotypes [26]. Overall, we demonstrated the validity of the functional sRNA transcriptomic approach employed in this work to find new virulence determinants in Mucorales.

## 3. Discussion

The RNAi mechanism plays an essential role in the defense against invasive nucleic acids and the regulation of endogenous transcripts [15,17,18,34]. The evolutionary conservation of these roles throughout the eukaryotic domain suggests a selective advantage to retain the mechanism of RNAi [35]. This observation is particularly highlighted in the case of Mucorales, a group of early diverging fungi that not only have conserved the canonical RNAi machinery but also have evolved a Dicer and Argonaute-independent noncanonical RNA mechanism and a special pathway involved in antifungal resistance not found in other organisms [6,21,34]. The RNAi mechanism regulates different pathways and cellular processes in *M. lusitanicus* [14]. Among these processes, virulence has been recently related to the RNAi mechanism in diverse regulatory pathways [22]. Here, we have developed an experimental approach to identify genes related to virulence and regulated by RNAi, showing as a proof of concept the identification and characterization of the gene *wex1*. Previous studies successfully tested a functional transcriptomic approach using samples from host—pathogen interactions, which resulted in long lists of candidate genes with putative roles in virulence [22,25]. That approach was pivotal to demonstrate the essential role of the RNAi pathways in virulence, controlling broad gene-networks during the host—pathogen interactions.

On the other hand, another study compared the Vi and Av pathotypes to find the differences that could explain their opposite virulence potential at a genomic level [26]. This genomic approach found 773 modified loci in the Av strain, which is also a long list of candidates for finding virulence factors. In this regard, our hypothesis contemplated comparing Vi and Av pathotypes but restricting the transcriptomic profiles to only those regulated by RNAi. To our knowledge, this work is the first application of a functional transcriptomic approach based on the differential production of sRNAs in virulent and avirulent strains.

Our specific transcriptomic analysis produced an approachable list of 75 candidate genes for roles in virulence and regulation by RNAi. The experimental validation of two randomly selected candidate genes demonstrated the reliability of this gene set. Moreover, the construction of avirulent-mimicking strains and their phenotypic study regarding virulence unveiled the essential role of the *wex1* gene in maintaining the virulence potential. The Vi strain ceases to be pathogenic when *wex1* is overpressed, showing a similar phenotype to the Av pathotype. Thus, the RNAi-dependant specific degradation of *wex1* mRNA levels in the Vi pathotype is required to ensure the virulence potential of this strain. The *wex1* gene encodes a putative DnaQ-like exonuclease, a superfamily of enzymes that catalyze nucleoside monophosphate excision at DNA or RNA ends in the 3′–5′ direction. These types of enzymes contain RNases domains such as RNase D and Rrp6p. A fine-tuned balance of exonuclease activity is critical for developmental processes and fungal virulence [36,37]. Assuming the hypothesis that *wex1* could be involved in the process of degradation and recycling of defective and aged mRNAs, the overexpression of this gene might reduce the levels of substrate mRNAs available for the noncanonical RNAi pathway (NCRIP) in *M. lusitanicus*. The competition for the target mRNAs among the different RNA degrading pathways has been proposed in several studies [21,23]. Thus, the overexpression of *wex1* would leave NCRIP without target substrate mRNAs, producing an avirulent strain similar to mutants lacking NCRIP activity [22]. This hypothesis could explain the same avirulent phenotype previously observed in NCRIP mutants and the strain overexpressing *wex1* generated in this study. However, other roles of *wex1* in unknown pathways related to virulence cannot be excluded at this point. 

The identification of *wex1* suggests that many others may be discovered by further dissection of our genes list. However, the functional characterization of the ARDL showed that this list also contains genes unrelated to virulence. Besides virulence, there are several differences between the Vi and Av pathotypes, such as mating type, heat stress tolerance, chitosan content and toxic compound susceptibility [26]. These differential phenotypes and their related cellular processes also define a close functional area in the fungal cell physiology complexity, which will facilitate the functional study of those candidates showing a phenotype unrelated to virulence. 

In summary, this work developed a successful approach to identify new virulence determinants regulated by the RNAi machinery. The sRNA transcriptomewide analysis of strains with opposite virulence potentials was functionally validated, presenting a narrowed list of 75 candidate genes that are both regulated by the RNAi machinery and related to the phenotypic differences between the two pathotypes. Among these candidate genes, the characterization of *wex1* and its essential role in virulence served as proof of concept for the entire approach. Moreover, the putative exonuclease activity of this gene might suggest a function in the complex interplay of the RNAi pathways in *M. lusitanicus* and their role in virulence. An additional advantage of the approach presented here is its exportability to any pathogen accounting with a conserved RNAi machinery and strains showing different pathogenic potential. Similarly, other model organisms having those two features can use our approach to investigate cellular processes unrelated to virulence. Further dissection of the genes identified here and the pathways in which they are involved will represent a new source of specific targets to design improved treatments against mucormycosis.

## 4. Materials and Methods

### 4.1. Fungal Strains

In summary, two different *M. lusitanicus* pathotypes were used as wild-type strains throughout the study: the avirulent pathotype NRRL3631 (hereinafter Av) and the virulent pathotype CBS277.49 (hereinafter Vi) [7,25,26]. Strain MU402 [27] derives from Vi and is a leucine and uracil double auxotroph that served as a recipient strain for all genetic transformations. As a result, mutant strains *wex1*∆ (MU641 and MU642), ARDL∆ (MU632), and P*zrt1*-*wex1* (MU637, MU638, MU639, and MU640) were generated. Double mutant strain in *dcl1* and *dcl2* was MU411 [28]. Unless otherwise stated, MU641, MU632, and MU640 were employed to conduct the experiments described in the manuscript.

### 4.2. RNA Isolation

We plated 2.5 × 10^5^ spores from Av and Vi strains in solid rich YPG medium with adjusted pH of 4.5 and incubated at a constant temperature of 26 °C for 24 h. Before spore inoculation, plates were covered with a thin layer of cellophane film to facilitate media-free mycelium harvesting after incubation. 100-mg strips of mycelium were harvested and used for RNA isolation. Sequencing sRNA samples were obtained with a mirVana miRNA isolation kit (Ambion, Thermo Fisher Scientific Inc., Waltham, MA, USA), whereas sRNA samples for Northern blotting were isolated employing Trizol and a polyethylene glycol-based differential precipitation of high molecular weight RNAs procedure described in previous studies [38]. The RNeasy plant Minikit (Qiagen, Hilden, Germany) was used to isolate mRNA following the supplier recommendations for fungal RNA.

### 4.3. RNA-Sequencing and Analysis for Small RNA Production

The sRNA samples from Av and Vi were sent to BaseClear sequencing facility. Libraries were prepared using TruSeq Small RNA Library Prep Kit and sequenced with a HiSeq 2500 to produce single-end, 50-bp reads. Raw sRNA reads from Av, Vi and publicly available *dcl1*∆ *dcl2*∆ double mutant strain (see Data Availability) were quality-checked with FASTQC v0.11.8, and adapter or contaminant sequences overlapping ≥2 bases at the 3′-end were removed with Trim Galore! v.0.6.2 (available at http://www.bioinformatics.babraham.ac.uk/projects/; accessed on 24 February 2021). Reads with Q ≤ 20 and total length ≤ 13 or ≥ 29 nt were excluded. Processed sRNA reads were aligned to the *M. lusitanicus* MU402 genome (from now on Muccir1_3, available at the JGI portal: https://mycocosm.jgi.doe.gov/Muccir1_3/Muccir1_3.info.html) using HISAT2 v2.1.0 [39]. The differential sRNA production between Vi and Av across all protein-coding loci was analyzed using DESeq2 v1.18.1 [40] and Muccir1_3 gene annotation. The resulting log_2_ fold-change (FC) and average log_2_ Counts per million (CPM) for sRNA production values were correlated in a scatter plot, highlighting three housekeeping genes that have a stable sRNA production among samples (EF-1 (Muccir1_3 ID: 1382517), TFIIIC (Muccir1_3 ID: 1386549), V-ATPase (Muccir1_3 ID: 1377858)). The z-score of log_2_ CPM values for Av, Vi and *dcl1*∆ *dcl2*∆ samples (calculated as the number of standard deviation units a value differs from the mean) was plotted using the pheatmap v.1.0.12 R package. The sRNA production values were clustered according to their similarity both at each protein-coding loci and sample using Euclidean distance and Ward’s clustering method. The average strand bias, i.e., the proportion of sense and antisense reads in all samples, was calculated for each locus plotted in the heatmap as the ratio comparing the difference of sense and antisense reads with all reads ((sense reads - antisense reads)/(sense reads + antisense reads)). Values close to 1 indicate a strong bias towards sense reads, whereas −1 implies a strong bias towards antisense reads; intermediate values (≈0) means an equal proportion of sense and antisense reads.

To generate sense and antisense sRNA genomic plots, aligned sRNA reads were split into forward and reverse strand reads by filtering through their Binary Alignment/Map (BAM) FLAG field (-F 16 for forwarding reads and -f 16 for reverse reads) with SAMtools v1.10-2 [41]. This allowed the identification of sense and antisense sRNAs as follows: forward or reverse reads aligning to same-sense protein-coding loci were considered sense sRNAs. Those aligning to opposite-sense protein-coding loci were considered antisense sRNAs. Aligned reads were normalized to bins per million reads (BPM) in 25-bp bins using deepTools v3.2.1 [42] bamCoverage function. Genomic plots were visualized using the deepTools pyGenomeTracks module.

### 4.4. Northern Blot Analyses for mRNA and sRNA

After sRNA isolation, samples were separated by denaturing urea polyacrylamide electrophoresis [43]. sRNAs with lengths ranging from 10 to 100 bp were transferred to a neutral Hybond-NX nylon membrane (Amersham. England, United Kingdom) and chemically fixed as described in other studies [38]. PCR-amplified fragments containing the target locus and the pT7 promoter sequence at their 5′ or 3′-end were used as DNA templates to transcribe in vitro specific sense or antisense riboprobes, respectively, using a MAXIscript™ T7 Transcription Kit (Ambion, Thermo Fisher Scientific Inc., Waltham, MA, USA). The [α-32P]UTP riboprobes were fragmented with an alkali solution, filtered to remove individual nucleotides, and hybridized to the transferred sRNAs as described in previous works [38]. The gel portion containing sRNAs with lengths ≥ 100 bp (5S rRNA and tRNAs) was stained with ethidium bromide as a loading control.

For mRNA Northern blotting, samples were separated by denaturing formaldehyde agarose electrophoresis and then transferred to a positively charged Hybond-XL nylon membrane (Amersham). Specific [α-32P]dCTP-labeled probes were generated with the Ready-To-Go (GE Healthcare Life Science) and used for hybridization. We hybridized 18S rDNA [α-32P]dCTP-labeled probes as loading controls. Both sRNA and mRNA membranes were developed using a phosphor screen (FUJIFILM Europe GmbH (Dusseldorf, Germany) and a Personal Molecular Imager system (Bio-Rad, Hercules, CA, USA) after a time exposure.

### 4.5. Genetic Transformation

Disruption fragments contained either a 2.0-kb fragment of the selectable marker *pyrG* for Ura+ or a 3.6-kb fragment of the selectable marker *leuA* for Leu+ selection, flanked by 1-kb upstream and downstream sequences of the target locus to facilitate gene replacement by homologous recombination. These fragments were PCR-amplified separately and then fused by overlapping PCR using specific primers for each deleted gene. MU402 was grown in solid rich YPG medium with adjusted pH 4.5 during 5–6 days for optimal growth and sporulation conditions. Spores were harvested to produce protoplasts as described in previous studies [38]. Protoplasts were transferred to 0.2 cm-cuvettes and electroporated with a field strength of 4 kV/cm (800 V), the capacitance of 25 µF, and a resistance of 400 Ω to allow the introduction of 5µg of disruption DNA fragments into the cells. After the pulse, the protoplasts were recovered in liquid YPG medium and plated into the selective medium. Protoplasts were plated in either a minimal medium with casamino acids (MMC) with adjusted pH of 3.2 to select Ura+ transformants, or yeast nitrogen base (YNB) minimal medium with adjusted pH of 3.2 to select Leu+ transformants. The transformants underwent 10 passages of vegetative sporulation, single-colony harvesting, and streaking in a selective medium to favor the selection of homokaryotic spores. Then, homokaryosis was confirmed by Southern blotting. Briefly, genomic DNA was purified as described in previous works [38], digested with specific restriction enzymes, and separated by electrophoresis. DNA fragments were transferred to a positively charged Hybond-XL nylon membrane (Amersham) and hybridized to specific [α-32P]dCTP-labeled probes, which were generated following the Ready-To-Go kit procedure (GE Healthcare Life Science). After stringent hybridization, the membranes were developed to discriminate mutant from wild-type DNA alleles, as described for Northern blotting.

### 4.6. Virulence Assays

Male OF-1 mice weighing 30 g (Charles River, Barcelona, Spain) were used as host models for virulence assays for their reliability in previous Mucoralean virulence assays [22,44]. The mice were immunosuppressed by intraperitoneal injection of cyclophosphamide (200 mg/kg of body weight), 2 days before the infection and once every 5 days thereinafter. Groups of ten were challenged intravenously by injecting 1 × 10^6^ spores in the retroorbital venous sinus [20]. The assay was done with two independent mutants overexpressing *wex1* (MU637 and MU638, Figure 5 and Figure S1, respectively). To ensure animal comfort, the mice were anesthetized with isoflurane via inhalation and monitored until they recovered from the anesthesia. Vi and Av strains were also injected as a positive and negative virulence control, respectively. Mice were housed under established conditions with free food and autoclaved water. Animal welfare was monitored twice a day for 20 days, and those mice meeting the criteria for discomfort were euthanized by CO_2_ inhalation. Survival rates during this time were plotted in a Kaplan—Meier curve, and differences were considered statistically significant with a *p*-value ≤ 0.05 in a Mantel—Cox test.

## Data Availability

The sRNA raw data files generated by this study are deposited at the National Center for Biotechnology Information Sequence Read Archive (NCBI SRA) and are publicly available through the project accession number PRJNA674566. These data were compared to a double *dcl1*∆ *dcl2*∆ mutant strain (accession number SRR039128) [14]. The *M. lusitanicus* genome Muccir1_3 [16] and annotation files can be accessed at the Joint Genome Institute (JGI) website (http://genome.jgi.doe.gov/; accessed on 24 February 2021) and used under the JGI Data Usage Policy.

## Supplementary Materials

The following are available online at https://www.mdpi.com/1422-0067/22/5/2282/s1.
