# Supplementary material for: The RNAi Mechanism Regulates a New Exonuclease Gene Involved in the Virulence of Mucorales
Source: Int J Mol Sci. 2021 Feb 25;22(5):2282. doi: 10.3390/ijms22052282 (PMC7956310; doi:10.3390/ijms22052282)
Supplement: Supplementary file 1 [file ijms-22-02282-s001.pdf]

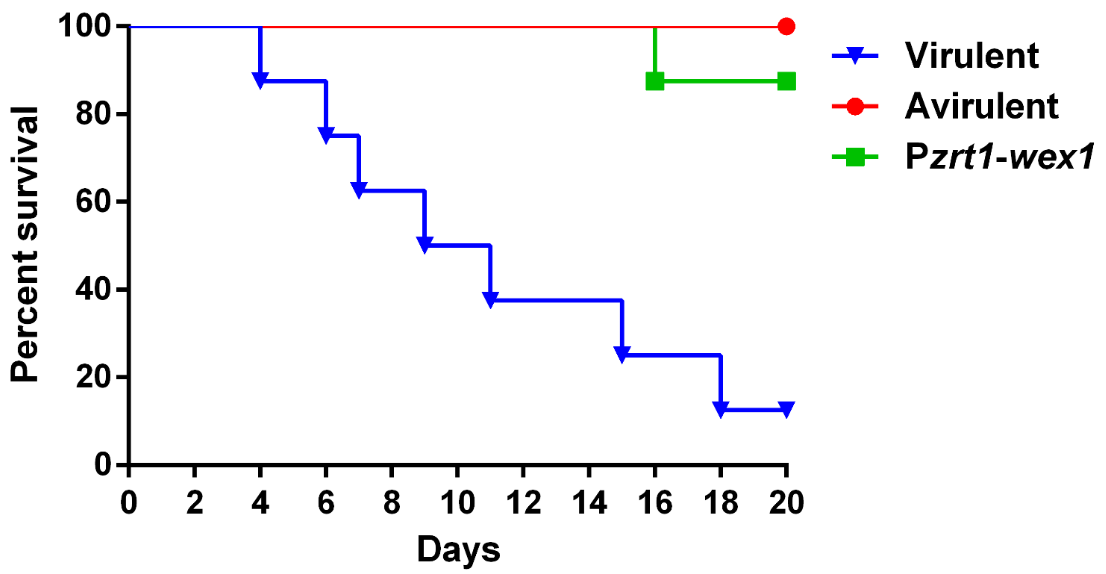

Figure S1. Second virulence assay testing the virulence of an independent mutant (MU638) overexpressing *wex1*. Mice were injected with $1x10^6$ spores of the P*zrt1*-*wex1* (green, MU638) mutant, the Vi pathotype (blue), and the Av pathotype (red). The survival rate of the mutant MU638 was compared to the Vi control strain and statistically analyzed by a Mantel-Cox test.
